# Colonic migrating motor complexes are inhibited in acute tri-nitro benzene sulphonic acid colitis

**DOI:** 10.1371/journal.pone.0199394

**Published:** 2018-06-22

**Authors:** Ben R. Hofma, Hannah R. Wardill, Chris Mavrangelos, Melissa A. Campaniello, David Dimasi, Joanne M. Bowen, Scott D. Smid, Claudine S. Bonder, Elizabeth A. Beckett, Patrick A. Hughes

**Affiliations:** 1 Adelaide Medical School, University of Adelaide, Adelaide, Australia; 2 Centre for Nutrition and GI Diseases, Adelaide Medical School, University of Adelaide and South Australian Health and Medical Research Institute, Adelaide, Australia; 3 Centre for Cancer Biology, University of South Australia and SA Pathology, Adelaide, Australia; University of Texas Medical Branch, UNITED STATES

## Abstract

**Background:**

Inflammatory Bowel Disease (IBD) is characterized by overt inflammation of the intestine and is typically accompanied by symptoms of bloody diarrhea, abdominal pain and cramping. The Colonic Migrating Motor Complex (CMMC) directs the movement of colonic luminal contents over long distances. The tri-nitrobenzene sulphonic acid (TNBS) model of colitis causes inflammatory damage to enteric nerves, however it remains to be determined whether these changes translate to functional outcomes in CMMC activity. We aimed to visualize innate immune cell infiltration into the colon using two-photon laser scanning intra-vital microscopy, and to determine whether CMMC activity is altered in the tri-nitro benzene sulphonic (TNBS) model of colitis.

**Methods:**

Epithelial barrier permeability was compared between TNBS treated and healthy control mice in-vitro and in-vivo. Innate immune activation was determined by ELISA, flow cytometry and by 2-photon intravital microscopy. The effects of TNBS treatment and IL-1β on CMMC function were determined using a specialized organ bath.

**Results:**

TNBS colitis increased epithelial barrier permeability in-vitro and in-vivo. Colonic IL-1β concentrations, colonic and systemic CD11b+ cell infiltration, and the number of migrating CD11b+ cells on colonic blood vessels were all increased in TNBS treated mice relative to controls. CMMC frequency and amplitude were inhibited in the distal and mid colon of TNBS treated mice. CMMC activity was not altered by superfusion with IL-1β.

**Conclusions:**

TNBS colitis damages the epithelial barrier and increases innate immune cell activation in the colon and systemically. Innate cell migration into the colon is readily identifiable by two-photon intra-vital microscopy. CMMC are inhibited by inflammation, but this is not due to direct effects of IL-1β.

## Introduction

Inflammatory Bowel Disease (IBD), incorporating Crohn’s Disease and Ulcerative Colitis, are chronic diseases characterized by overt inflammation of the lower gastrointestinal (GI) tract. IBD is frequently accompanied by symptoms of diarrhea, abdominal pain and cramping, indicating that inflammation alters enteric neuronal function in the colon. Somewhat paradoxically, colonic motility has consistently been observed to slow during IBD both in humans and in animal models of colitis [1, 2]. Molecular studies have revealed how the chemical coding and electrical properties of enteric neurons are altered in animal models of colitis [1, 2]. However, the effect of inflammation on colonic migrating motor complexes remain to be determined.

Rectal enemas of tri-nitrobenzene sulphonic acid (TNBS) have long been used to model the inflammatory aspects of colitis [3, 4]. We, amongst others, have recently demonstrated that the acute stage of TNBS colitis is associated with activation of innate immune responses, which results in infiltration of macrophages into the colon and increased concentrations of key innate cytokines including IL-1β [5]. Innate immune cells, including granulocytes and monocytes, are distinguished from other immune cell lineages by the extracellular marker CD11b. These cells largely originate in the bone marrow and migrate via the blood system to reside in tissues. However, the spleen is also an important reservoir of innate immune cell precursors, particularly monocytes [6], and the relative importance of these sites toward innate immune cell activation during TNBS colitis remains unknown. Recent advancements in two-photon laser scanning microscopy have enabled visualization of cell migration in deep visceral tissues including the liver and small intestine [7, 8], but are yet to be applied to the inflamed colon. In the following experiments we demonstrate that intra-vital microscopy readily identifies cells that are migrating from colonic blood vessels into the colon, providing a useful tool for evaluating immune cell infiltration into colon *in-vivo* and in real time.

The movement of colonic luminal contents over long distances is directed by the Colonic Migrating Motor Complex (CMMC). CMMCs comprise of long lasting contractions of the colonic musculature that propagate aborally and are governed by a complex interaction between colonic smooth muscle and the enteric nervous system, with myenteric neurons in particular playing a key role [9–12]. Colonic motility has previously been shown to decrease during chemically induced colitis [13–16], an effect attributed to a suppression of both excitatory (e.g. cholinergic) and inhibitory (e.g. nitrergic) signaling in the colon [16–18], and a loss of myenteric neurons [15, 19–22]. Innate immune activation and the key innate immune cytokine IL-1β have previously been shown to both upregulate the early activated gene product c-fos on colonic myenteric neurons, and also alter the function of cholinergic and nitrergric colonic myenteric neurons [23, 24]. However, little is understood regarding the impact of colitis and IL-1β on CMMC activity.

We aimed to develop a protocol for two-photon laser scanning microscopy of inflammation in the colon, and to determine the effects of colitis on CMMC activity.

## Materials and methods

All experiments were approved by the Animal Ethics Committees of The University of Adelaide and SA Pathology and performed according to the N3CRs Arrive guidelines (S1 Table). Animals were sourced from the Animal Resource Centre, Canning Vale, Western Australia. Animals were conventionally housed with *ad libitum* access to standard diet and tap water and were euthanized by overdose of inhalation CO_2_, except for intravital experiments where already anaesthetized mice (ketamine / xylazine–see below for details) were cervical dislocated at the end of the experiment. All experimental procedures were initiated between 9 and 11 am. Experiments were restricted to male mice to negate the potential confounding effects of estrous cycle.

### TNBS colitis model

20-30gm C57/Bl6 male mice were anaesthetized (isoflurane; 2–4% in oxygen) and 0.1ml TNBS (130μl of 1M TNBS solution in 30% EtOH / ml) or 0.1ml 30% EtOH alone (vehicle) instilled into the colon via a polyethylene catheter inserted 3 cm from the anus, after which mice were held upside down for 1 minute [5, 25–27]. TNBS treated mice were provided with post-operative care (head pad, *ad libitum* access to soaked food) and allowed to recover for 2 days. Mice were weighed prior to 10am each day. Colon length was measured on excised colons dissociated free from surrounding tissue from the tip of the mouse anus to the distal end of the cecum.

### Ussing chamber

The whole colon was removed and flushed with cold phosphate buffered saline (PBS). Segments of the distal colon were immediately mounted into Ussing Chambers (Physiologic Instruments, California, USA) for electrophysiological analyses as previously described [28]. Briefly, the colon was cut longitudinally along the mesenteric attachment and placed onto a 0.1 cm^2^ aperture sliders. The tissue was mounted and continually bathed in an oxygenated, glucose-fortified Ringers solution (composition in mmol/L/L: NaCl 115.4, KCl 5, MgCl_2_ 1.2, NaH_2_PO_4_ 0.6, NaHCO_3_ 25, CaCl_2_ 1.2, and glucose 10). Tissues were voltage clamped to zero potential difference by the application of short-circuit current (I_SC_) and allowed to equilibrate for 20 min before baseline I_SC_, conductance (S/cm^2^) and trans-epithelial electrical resistance (R_TE_; Ω.cm^2^) were averaged over a 10 min time period.

### FITC-dextran translocation

3 h prior to kill time-points mice were gavaged orally with a 500 mg/kg dose of 4-kDa FITC-dextran (75 mg/mL; Sigma). Blood was collected via cardiac puncture into Multivette 600 Serum-Gel with Clotting Activator capillary tubes (Sarstedt, SA, Australia) and stored on ice for 30 minutes. Serum was isolated by centrifugation (10,000 × *g* 10 min. at room temperature), diluted 1:3 with PBS and absorbance read at 450nm. FITC-dextran concentrations were determined based on relationship to standards of known concentration.

### ELISA

For enzyme-linked immunosorbent assay (ELISA) a 1cm section of distal colon was removed, snap frozen in liquid nitrogen and stored at -80°C before homogenization in cell extraction buffer (Thermo-Fisher Scientific, NSW, Australia) supplemented with 1mM phenylmethanesulfonyl fluoride (PMSF) (Sigma) and protease inhibitor cocktail (Sigma) at 1ml per 50mg tissue as previously described [5]. Supernatants were collected and stored at -80°C until analysis. Total protein concentration was calculated by BCA assay (Abcam, UK) as previously described [5]. IL-1β concentration was determined by ELISA (OptiELISA, BD Biosciences, NSW, Australia) essentially as described in the manufacturer’s instructions. 96 well flat bottom plates (Nunc-Immuno, Thermo-Fisher Scientific) were coated with α-IL-1β capture antibody overnight at 4°C and blocked at room temperature for 1 hour in Assay Diluent. Samples and standards were added for 2 hours, after which detection antibody (biotin α-IL-1β) was added for 1 hour, then Avidin-HRP for 30 minutes and finally substrate solution (Tetramethylbenzidine / Hydrogen Peroxide) for 30 minutes, all at room temperature. The reaction was then stopped and absorbance read at 450nm. Total protein and IL-1β concentrations were determined based on relationship to standards of known concentration. The limit of sensitivity was <13 pg/ml.

### Flow cytometry

Spleen and bone marrow were dissected free from surrounding tissue and mechanically dissociated into suspensions and filtered over a 40μM strainer (Corning, NY, USA). Blood was collected by intra-cardiac puncture into heparinised tubes. Colonic lamina propria mononuclear cells (LPMC) were isolated enzymatically as previously described [5]. Briefly, 1mm colon pieces were incubated in Hepes buffered HBSS supplemented with 1mM EDTA and 1mM DTT (Sigma) for 20 min. at 37°C under slow rotation and passed through a 100μM cell strainer. The residual tissue was minced and incubated for 20 min at 37°C in complete media (RPMI 1640 (Gibco, Germany) supplemented with fetal calf serum, glutamax and penicillin / streptomycin) with 1mg/ml Collagenase D (Roche, NSW, Australia), 0.5mg/ml DNAse1 (Sigma) and 3mg/mL Dispase (Roche) added. LPMC suspensions were passed through a 40μM strainer and repeated until no tissue remained. The flow through was centrifuged (500g, 10 min.) and the pellet resuspended over a 40/80 Percoll (Sigma) layer, centrifuged (1000g, 20 min.) and interphase layer collected and centrifuged in PBS (500g, 10 min). Spleen, bone marrow, blood and LPMC suspensions were stained with trypan blue to determine viability [5, 27]. Cell number and viability were determined using an automated counting system (Countess, Invitrogen, CA, USA).

0.5 x 10^6^ F_c_ blocked (BD Biosciences), LPMC, bone marrow, and spleen cells were stained for viability (FVS700, BD Biosciences) and the following anti-mouse monoclonal antibodies: CD45-BV605, CD326-BV510, CD3-BUV395, CD11b-APCCy7 (BD Biosciences). 50 μL of fresh blood was stained with the above antibodies prior to red cell lysis by incubating in red blood cell lysis buffer (BD Bioscience) for 5 min at room temperature. Immediately prior to analysis, 50μL of Trucount beads (BD Bioscience) were added to each tube for analysis of absolute numbers. 20,000 events / tube were analysed on a LSR FORTESSA X-20 flow cytometer (BD Biosciences) and proportions of live singlets were determined using FlowJo (Tree Star, OR, USA) as previously described [27].

### Intravital microscopy

Mice were anaesthetized by intraperitoneal (i.p.) injection of ketamine (10% v/v, Ceva Group, NSW, Australia) and xylazine (5% v/v, Ceva Group) in 0.9% saline, and 1mg kg^-1^ atropine was administered i.p. to reduce peristaltic contractions. 100μL fluorescent dye solution containing 150 kDa FITC-dextran (Sigma) with CD11b-AF 594 (3μg / 100 μL PBS (Biolegend, CA, USA)) or Rhodamine 6G (1mg 100μL^-1^ PBS (Sigma)) was administered intravenously via an intraorbital injection. The colon was then exposed by a simple midline laparotomy with care taken to minimize bleeding. If bleeding occurred the area was immediately cauterized. The colon was then raised onto a purpose-built stage, cover-slipped and positioned under a 20x objective and 10X eyepiece within a heated chamber of a LSM-710-NLO 2-photon microscope (Carl Zeiss, Jena, Germany). FITC-dextran and CD11b-AF594 were excited using a Mai-Tai Ti:Sapphire multiphoton laser (Spectra-Physics, Santa Clara, CA) and external non-descanned detectors were used to capture the fluorescence signal [29]. Live images were recorded every 500msec. for 3 minutes using Zen 2011 (version 7.0.4.0, Carl Zeiss). Video images were analyzed off-line by a blinded researcher with adherent leukocytes defined as those remaining stationary for >30secs.

### Colonic migrating motor complex activity

The entire distal portion of the gastrointestinal tract (from anus to cecocolic junction) was isolated from healthy or TNBS treated mice and flushed with Krebs solution containing (in mM): NaCl 118.0; KCl 4.75; NaHCO_3_ 25.0; glucose 11.0; MgSO_4_ 1.2; NaH_2_PO_4_ 1.0; CaCl_2_ 2.5) to remove fecal material. The most distal 1 cm of rectum was discarded before securing the remaining large bowel, via entomology pins through the mesenteric attachments, to the Sylgard base of a specialized organ bath. High sensitivity tension transducers (MLT0202, AD instruments, Sydney) were attached via suture thread to distal, mid and proximal sites (located 20 mm, 55 mm and 95 mm from anus, respectively) via small forked steel hooks through the smooth muscle [10]. Colonic tissue was superfused with Krebs with pH maintained at 7.3–7.4 when bubbled with 95% O_2_ / 5% CO_2_ at 36.0 ± 0.5°C. Passive tension of 10mN was applied via each recording hook and colons were allowed to equilibrate for 30 minutes before experimentation. Analogue signals from the tension transducers were digitized (Powerlab ML866 and quad bridge amp ML221, AD instruments, NSW, Australia) and relayed to a PC running LabChart Pro 8 (AD Instruments). In some experiments 10 ng ml^-1^ IL-1β (Sigma) was added to the organ bath Krebs for 30 min. Time control experiments were performed for 30 min with Krebs alone. CMMC duration, amplitude and frequency were analyzed off-line from the digital traces. Frequency, amplitude and duration of coordinated peaks were assessed from the using established experimental parameters [30]. Frequency was determined by counting the number of events that occurred during a 20 minute period. Amplitude was determined as the peak tension minus baseline. The duration of each contraction was determined as the time between the half-maximal-amplitude points on the rising and falling phases.

### Statistical analysis

In all cases results are expressed as Mean±SEM. Unpaired Student’s t-tests determined the significance of changes observed between healthy control and TNBS except for body weight changes where Two-way ANOVA with Tukey’s post-hoc was used. The significance of effects of IL-1β and time-control on CMMC activity were determined by paired Student’s t-test.

## Results

### TNBS colitis is characterised by wasting, reduced colon length, epithelial damage and increased colonic IL-1β

**Fig 1:**
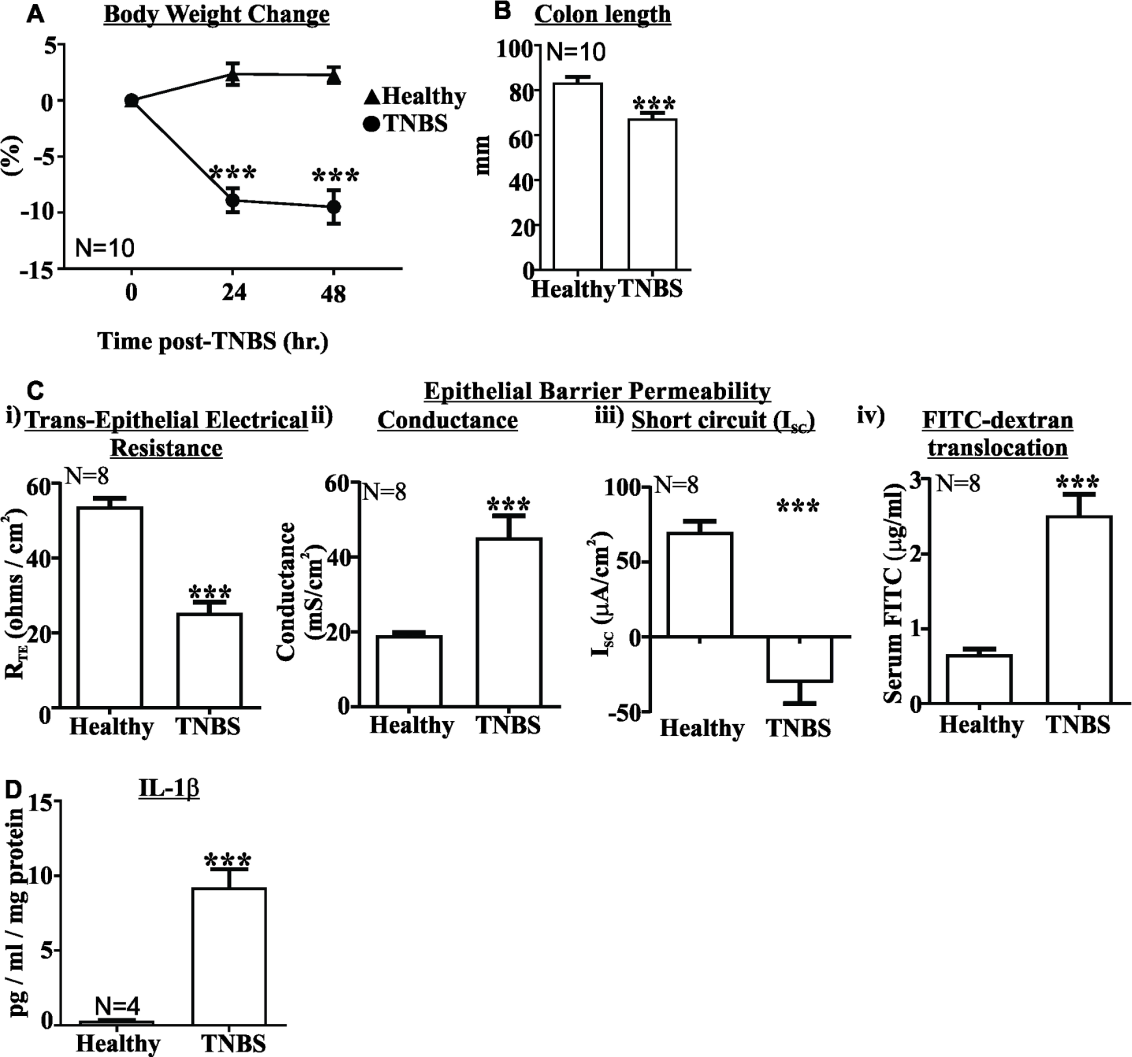
TNBS colitis reduces body weight, shortens colon length, damages epithelial barrier integrity and increases colonic IL-1β. A) Body weight was significantly reduced 24 and 48 hours following TNBS treatment relative to healthy controls (▲ Healthy, ● TNBS, N = 10). B) Colon length was significantly shortened 48 hours following TNBS treatment relative to healthy controls (N = 10). C) The epithelial layer is damaged in TNBS colitis, with i) increased trans-epithelial electrical resistance (R_TE_), ii) decreased epithelial conductance, iii) decreased epithelial short-circuit current (I_SC_), and iv) increased FITC-dextran translocation into serum. D) IL-1β concentrations are increased in TNBS colitis. (N = 4). Mean±SEM. *** P<0.001.

Body weight was reduced by approximately 10% 48 hours following TNBS administration, which was significantly lower than untreated healthy controls (P<0.001, Fig 1A). Colon length, used as an indication of overt inflammation, was also shortened by approximately 20% in TNBS treated mice relative to healthy controls (P<0.001, Fig 1B), as previously observed [5, 25, 26]. The integrity of the epithelial layer was damaged in the distal colon of TNBS treated mice with reduced trans-epithelial electrical resistance (R_TE_), increased conductance and reduced short circuit current (Fig 1Ci, 1Cii and 1Ciii respectively). This was also associated with a pronounced increase in the serum concentration of gavaged 4kDa FITC-dextran, indicating that our *in-vitro* permeability findings translate *in-vivo* (Fig 1Civ). Treatment with ethanol vehicle had no effect on in-vitro or in-vivo measurements of epithelial permeability (S1 Fig), as previously demonstrated with MPO measurements [5]. Colonic concentrations of IL-1β, the signature innate immune cytokine, were substantially increased in TNBS treated mice (P<0.001, Fig 1D) relative to healthy controls, as previously observed [5].

### Innate immune cell infiltration in colitis

**Fig 2:**
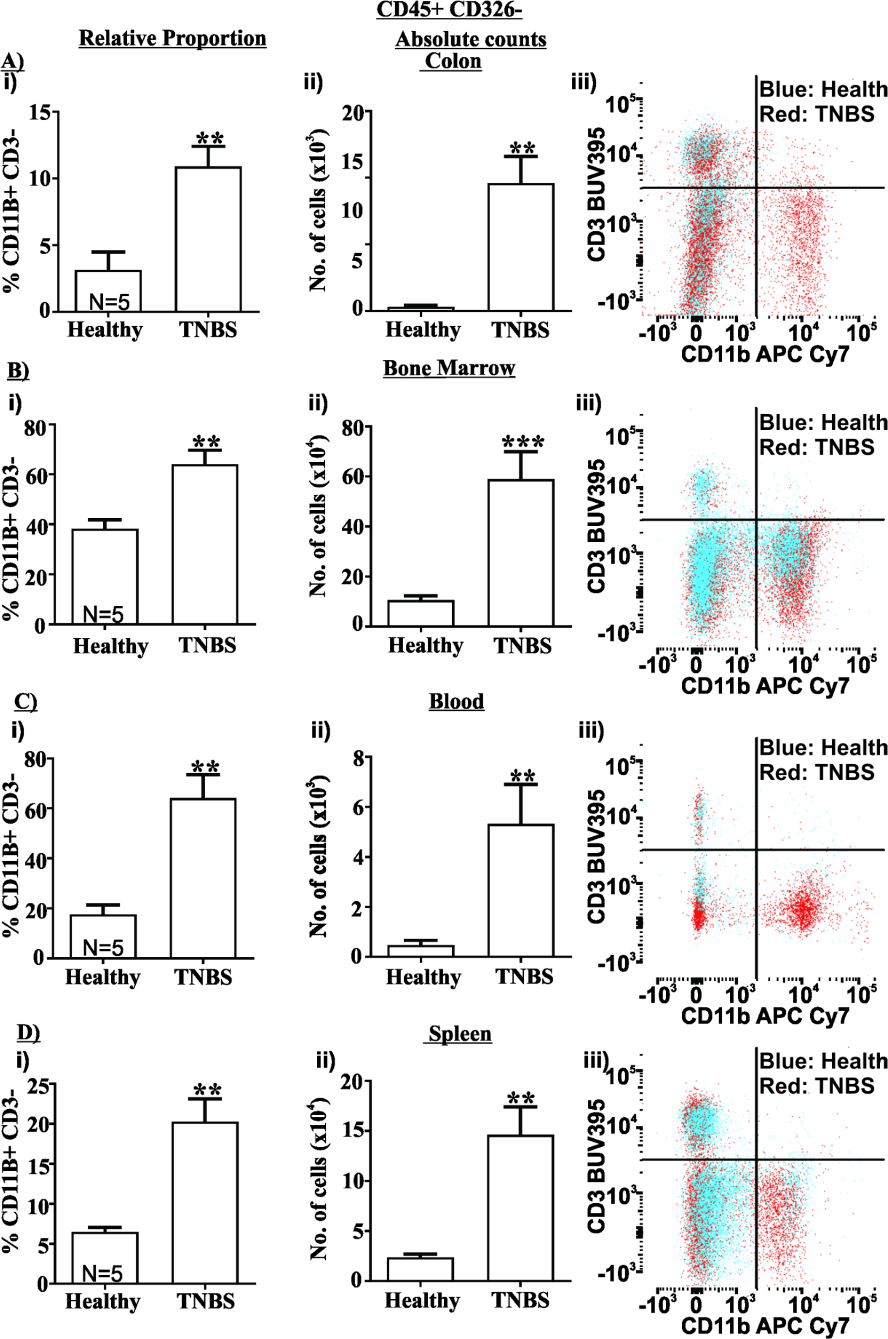
TNBS colitis causes innate cell infiltration into the colon, and expansion in the bone marrow, blood and spleen. The relative proportion (i) and absolute number (ii) of CD45+ CD326-, CD11b+ CD3- innate cells increased in the colon (A), bone marrow (B), blood (C) and spleen (D) 48 hours after TNBS treatment relative to healthy controls (N = 5 / group). Also shown are representative flow cytometry plots of CD11b APCCy7 / CD3 BUV395, where blue is healthy controls and red is TNBS treated (Aiii-Diii). Mean±SEM. ** P<0.01, *** P<0.001.

We observed an approximately 3.5-fold increase and a 30-fold increase in the relative proportion and absolute number of CD11b+ CD3- innate immune cells in TNBS treated mice relative to healthy controls (P<0.01 for both comparisons, Fig 2Ai (relative proportion) and 2Aii (absolute number)). Similar results were also observed in the bone marrow with a 1.5-fold increase in the relative proportion and a 5.5-fold increase in absolute number of innate immune cells (P<0.01 and P<0.001 respectively relative to healthy controls, Fig 2Bi and 2Bii). Interestingly, we also observed a substantial increase in innate immune cells in both the blood (3.5-fold increase in relative proportion and 11-fold increase in relative number, P<0.01 for both, Fig 2C) and in the spleen (3-fold increase in relative proportion and 6-fold increase in absolute number, P<0.01 for both, Fig 2D), indicating the systemic circulation is involved in the inflammatory response in TNBS colitis.

**Fig 3:**
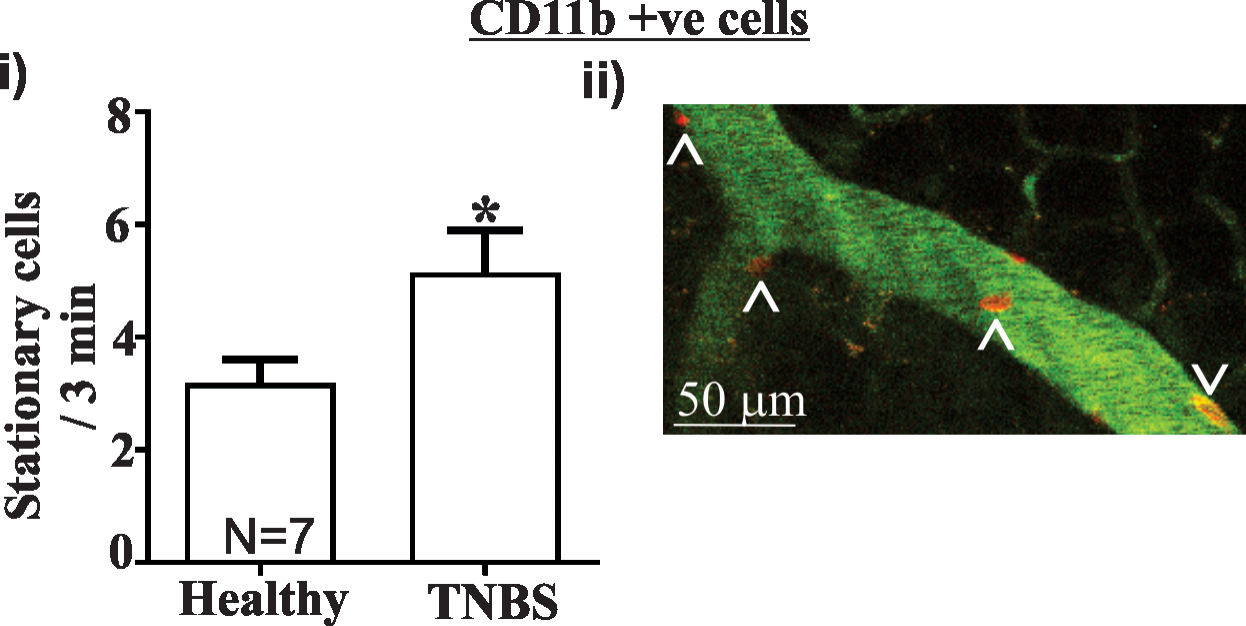
TNBS colitis causes an increase in the number of stationary CD11b+ cells in colonic blood vessels and IL-1β concentration. i) Intravital imaging of colonic blood vessels revealed that an increased number of CD11b AF-594 +ve cells were stationary 48 hours following TNBS treatment relative to healthy controls (N = 7). ii) Representative image of stationary (Λ) and moving (V) cells in colonic blood vessels containing FITC-Dextran. Still image adapted from video imaging (See S2 and S3 Figs for videos). Scale bar = 50 μm. Mean±SEM. * P<0.05.

Intravital imaging revealed that the number of stationary CD11b+ cells was increased more than 1.5 fold in 150kDa FITC-Dextran labelled blood vessels within the colon wall of TNBS treated mice relative to healthy controls (P<0.05, Fig 3; See S2 and S3 Figs for video images). Stationary cells are the cells that are migrating to and from tissue and are therefore integral to the immune response observed in the colon.

### Enteric neurons are in close proximity to blood vessels in the colon wall

**Fig 4:**
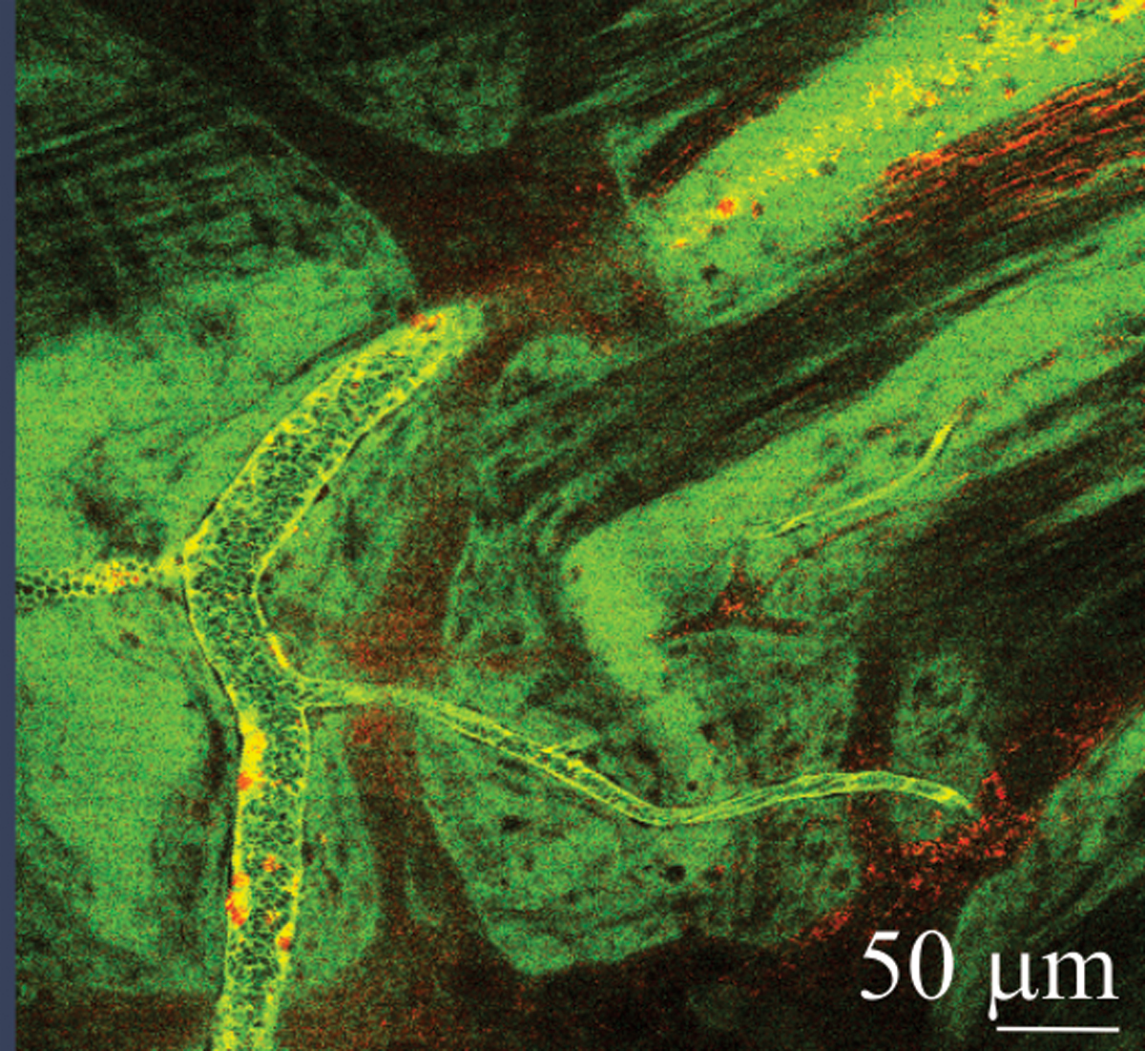
Blood vessels are in close apposition to enteric neurons in the colon. Still image adapted from video file demonstrating the close apposition of Rhodamine 6G labelled enteric neurons to FITC-dextran containing blood vessels in the colon (see S4 Fig for video).

Some mice were also administered the non-specific nuclear label Rhodamine 6G instead of CD11b-AF594, which revealed the close proximity of enteric neurons to blood vessels (Fig 4, see S4 Fig for video), and therefore the potential for immune mediators to influence enteric neuronal behaviour.

### Effects of colitis on CMMC activity

**Fig 5:**
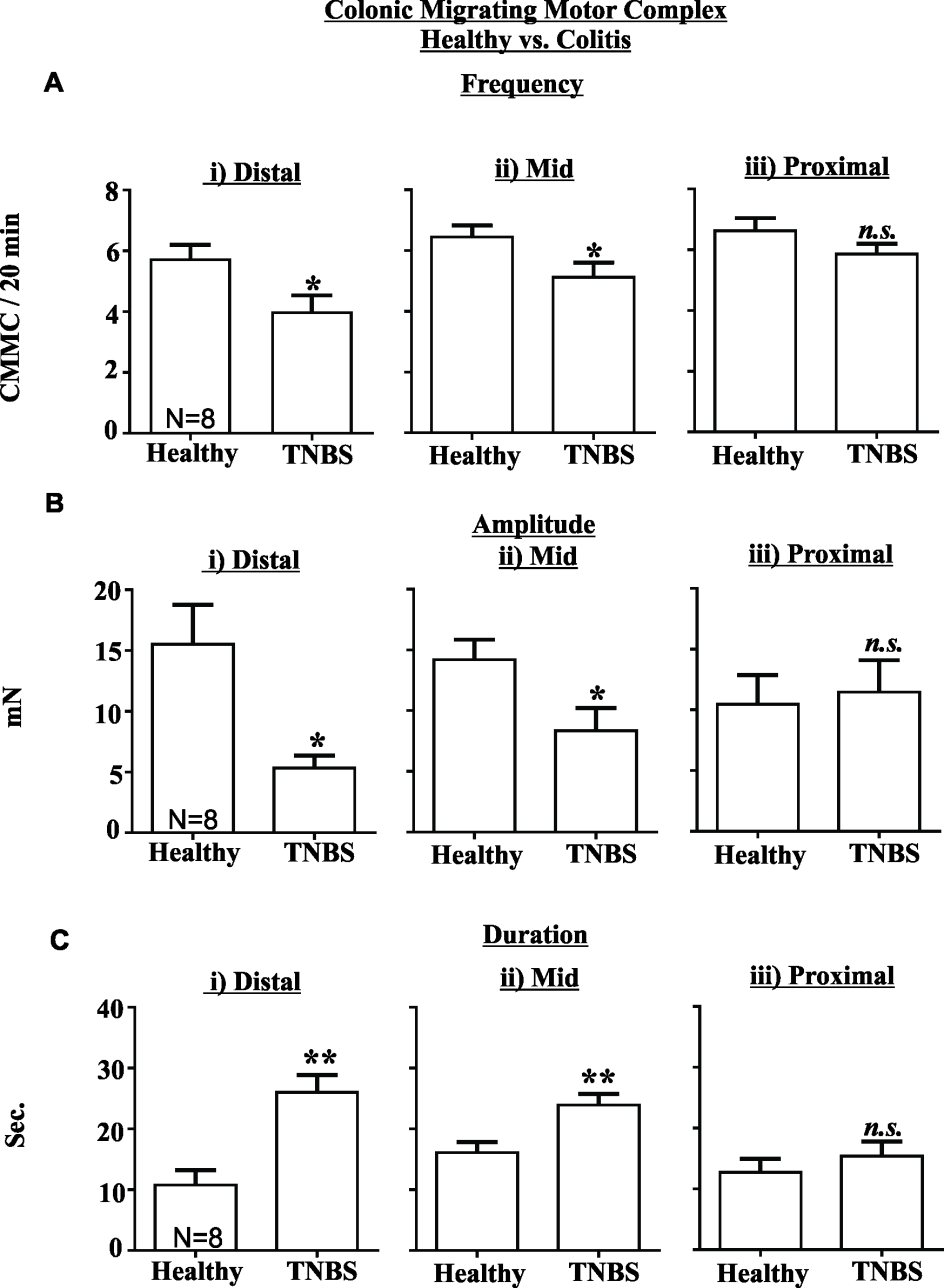
TNBS colitis inhibits colonic migrating motor complexes (CMMC) in the distal and mid colon. The frequency (A) and amplitude (B) of CMMC were reduced in the (i) distal and (ii) mid colon, but were unchanged in the (iii) proximal colon 48 hours following TNBS treatment relative to healthy controls. (C) The duration of CMMC was increased in the (i) distal and (ii) mid colon, but unchanged in the proximal colon 48 hours after TNBS treatment relative to healthy controls. (N = 8). Mean±SEM. * P<0.05, ** P<0.01.

**Fig 6:**
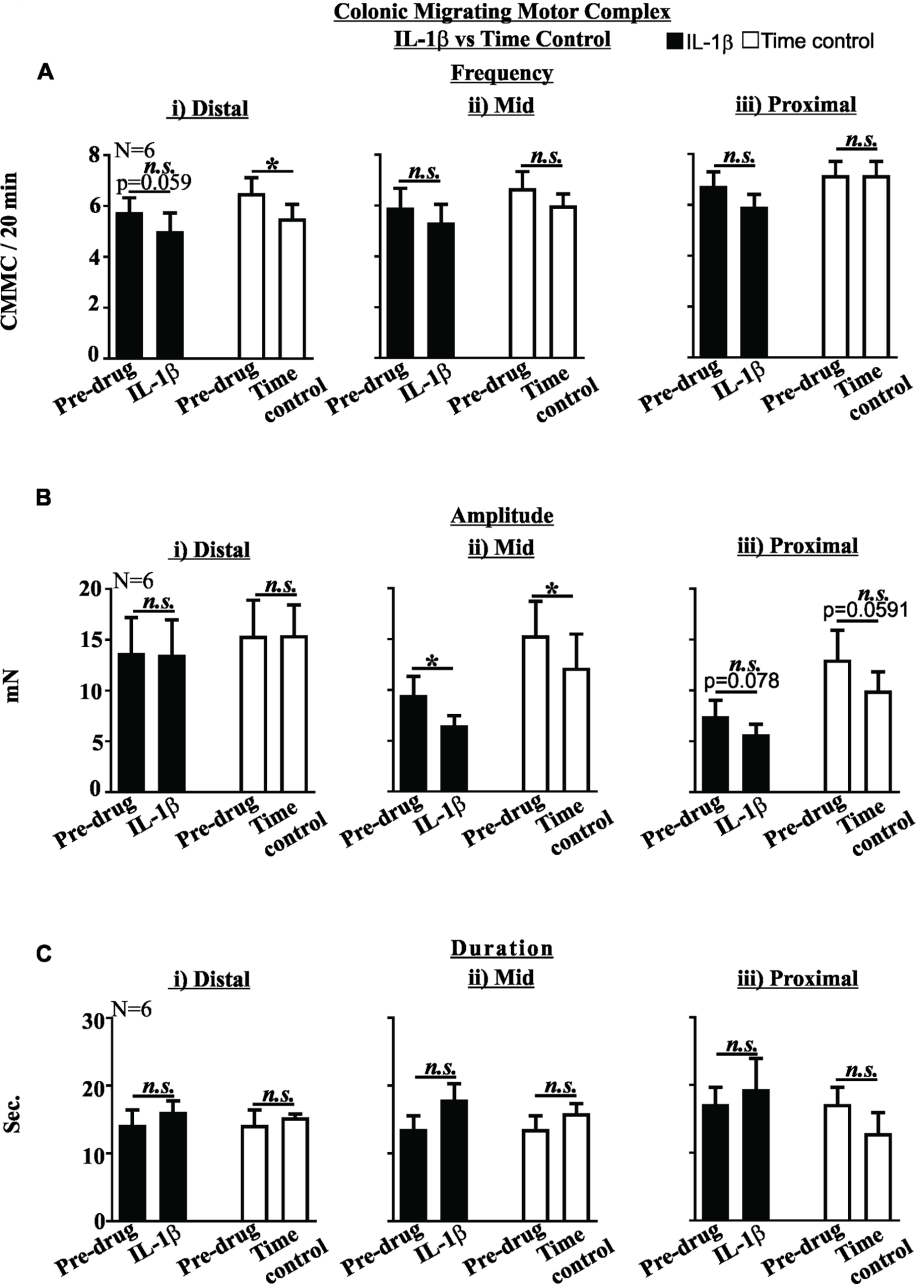
IL-1β does not alter colonic migrating motor complex activity. The effects of 10ng/ml IL-1β (full bars) on CMMC frequency (A), amplitude (B) and duration (C) in the distal (i), mid (ii) or proximal (iii) colon did not differ from time controls (clear bars). (N = 6). *n*.*s*. not significant. Mean±SEM. * P<0.05.

TNBS treated mice displayed reduced CMMC frequency in both the distal (P<0.05, Fig 5Ai) and mid- colon (P<0.05, Fig 5Aii), reduced CMMC amplitude in both the distal (P<0.05, Fig 5Bi) and mid-colon (P<0.05, Fig 5Bii) and increased CMMC duration in both the distal (P<0.01, Fig 5Ci) and mid-colon (P<0.01, Fig 5Cii) compared to healthy controls. There was no significant difference between the magnitude of these effects between distal and mid- colon (data not shown). CMMC frequency, amplitude or duration in the proximal colon were not affected by TNBS colitis (Fig 5Aiii, 5Biii and 5Ciii). Perfusion of healthy control colon tissue with IL-1β (10 ng/ml) did not alter CMMC frequency, amplitude or duration relative to time control experiments (Fig 6).

## Discussion

IBD is characterised by overt inflammation of the intestinal tract and is often accompanied by bloody diarrhea. Human and animal models consistently demonstrate that colonic motor function is altered in IBD, however the actions of inflammation on colonic motor function remain to be definitively determined. Here we demonstrate that epithelial damage and innate immune cell infiltration into the colon in acute TNBS colitis is associated with increased concentrations of the key innate immune cytokine IL-1β and pronounced inhibitory effects on colonic motor activity.

IBD is typically accompanied by diarrhoea, however, and somewhat in contradiction, colonic motor activity in response to a meal is inhibited in human subjects with active IBD [31]. CMMC comprise the rhythmic contractions that propel faecal contents toward the anus. They are governed by a complex interaction between enteric neurons and smooth muscle [9–12]. In our current study we observed that CMMC activity is reduced during acute TNBS colitis, in line with observations made in humans and other studies investigating the molecular mechanisms underlying colonic dysfunction in TNBS colitis (reviewed in [1, 2]). The decrease in CMMC frequency and amplitude but increase in duration in inflamed regions indicates that CMMC are reduced in number and have weaker contractile force but occur over a longer time period, implying that motility is inhibited in these regions. Intensive research over the last couple of decades has provided important insights into the mechanisms underlying CMMC generation and propagation [32]. Detailed electrophysiological recordings from enteric neurons and colonic smooth muscle indicate that the inhibition of colonic motility in TNBS colitis involves both an increase in AH neuron excitation and a reduction in descending neuromuscular inhibition, but only in inflamed regions [13, 14, 16]. Our current study confirms these findings as the reductions in CMMC activity were only observed in the distal and mid colon of TNBS treated mice, and not in the unaffected proximal colon.

TNBS colitis is characterised by a wasting behaviour that involves innate immune responses in its acute stage [4, 5]. Two days after TNBS administration we observed signs of overt inflammation including reduced body weight, shortened colon length, and a substantial loss of epithelial barrier integrity, confirming observations that we, amongst others, have previously made in this model [5, 25, 26, 33]. We also observed a striking increase in the infiltration of CD11b+ cells into the colon. CD11b is a pan-innate immune cell marker that is expressed on the cell surface of monocyte / macrophages, natural killer cells and granulocytes such as neutrophils. The bone marrow constitutes an important source of innate immune cells, and we observed a significant increase in both the relative proportion and absolute number of CD11b+ cells in the bone marrow of colitic mice. Interestingly, we also observed substantial increases in CD11b+ cells in the spleen and in circulating blood, indicating that TNBS colitis also involves a systemic immune response.

We used intra-vital imaging coupled with 2-photon microscopy to assess the migration of CD11b+ cells in blood vessels in the colon. This powerful technique permits visualisation of immune cell movement *in-vivo* and in real-time as recently observed in investigations of immune activation in allergic responses, stroke and antigen processing in the small intestine [7, 8, 29]. To the best of our knowledge, this is the first application of 2-photon intra-vital microscopy in the colon of colitic mice. We observed that a greater number of CD11b+ cells were stationary in blood vessels in colitic mice compared to health. In this setting, stationary cells are undergoing extravasation, moving from blood vessels into the site of inflammation, and these findings confirm our evidence from cytometry that CD11b+ cells are increased in the colon of colitic mice. Interestingly, we also observed blood vessels to be in very close apposition to enteric neurons, indicating immune cells and secreted mediators are likely to influence the behaviour of enteric neurons.

Innate immune activation is a hallmark of IBD, as evidenced by the clinical utility of measuring faecal calprotectin. IL-1β is a signature innate immune cytokine predominately secreted by neutrophils and monocyte / macrophages. We confirm that tissue concentrations of IL-1β are increased in colitic mice, as previously observed [5]. Molecular studies have previously demonstrated that IL-1β has prominent effects on neuronal activity including upregulating Fos expression, suppressing cholinergic activity in colonic myenteric neurons, exciting ileal submucosal neurons and activating nociceptive colonic extrinsic sensory afferent endings [23, 24, 34, 35]. Combined these results indicate IL-1β may have a role in colonic disease states including dysmotility, diarrhoea and lower abdominal pain. However, we previously observed that incubation of human colonic resections with IL-1β did not affect cholinergic contractility of longitudinal muscle strips [36]. Here we superfused healthy colonic tissue with IL-1β at a concentration consistent with what we have previously used to excite colonic sensory afferent nerves and similar to the whole colon concentration we measured in TNBS colitic mice. We observed that IL-1β did not significantly alter CMMC activity with respect to the normal run-down observed in time-control experiments. Importantly, cytokines including IL-6 and TNF-α are also increased in TNBS colitis, are known to alter colonic nerve function and may also contribute toward altered CMMC [5, 37–39].

In conclusion, we observed that innate immune responses are activated and CMMC activity is reduced in acute TNBS colitis. The decrease in CMMC activity caused by colitis does not involve a direct effect of IL-1β. These results indicate that targeting the innate immune system, and IL-1β in particular, may alleviate inflammatory aspects involved in colitis. However, any beneficial effects that an approach targeting IL-1β has on motility would be secondary to a reduction in inflammation.

## Supporting information

S1 Fig Vehicle (Ethanol) treatment had no effect on in-vitro or in-vivo measurements of epithelial permeability including i) epithelial resistance, ii) epithelial conductance, iii) epithelial short-circuit current, and iv) FITC-dextran translocation into serum.
(TIF)

S2 Fig Intravital microscopy video of CD11b+ cells in FITC-dextran labelled colonic blood vessels in healthy mice. (AVI)

S3 Fig Intravital microscopy video of CD11b+ cells in FITC-dextran labelled colonic blood vessels in TNBS mice. (AVI)

S4 Fig Intravital microscopy video of rhodamine-6G labelled enteric neurons and immune cells in FITC-dextran labelled colonic blood vessels. (AVI)

S1 Table NC3RS ARRIVE guideline checklist. (PDF)
